# Assessment of the Retinal Ganglion Cell Layer after Uncomplicated Cataract Surgery

**DOI:** 10.3390/jcm13123579

**Published:** 2024-06-19

**Authors:** Bassam Abou-Jokh Rajab, Carlos Doncel-Fernández, Noelia Sánchez-Liñan, Gracia Castro-Luna

**Affiliations:** 1 Department of Ophthalmology, Hospital Universitario Poniente, 04700 Almería, Spain; b_jokh@hotmail.com (B.A.-J.R.); carlosdoncel@hotmail.com (C.D.-F.); 2 Department of Nursing, Physiotherapy, and Medicine, University of Almeria, 04120 Almería, Spain; noeliaszlszl@gmail.com

**Keywords:** ganglion cell layer, cystoid macular edema, central macular thickness, cataract surgery

## Abstract

(1) **Background:** This research aimed to evaluate the changes in ganglion cell layer thickness (GCLT) after uncomplicated cataract surgery in patients without previous ocular pathology and the impact of the appearance of cystoid macular edema on the GCLT and visual acuity. (2) **Methods:** The evaluation of 174 patients was performed with the indication of uncomplicated cataract surgery. The variables analyzed were demographic data, best-corrected visual acuity (BCVA), cataract type and OCT (Optical Coherence Tomography) measurements of central macular thickness (CMT), and the presence of cysts and GCLT preoperatively and one day, one and three months after surgery. (3) **Results:** There was a relationship between the postoperative increase in retinal GCLT and BCVA after uncomplicated cataract surgery. The presence of microcysts reduced the thickness of the GCL, which is significantly related to the loss of BCVA. The appearance of cystoid macular edema one month after surgery was also related to the preoperative CMT. There was a statistically significant decrease in preoperative GCL but a statistically significant increase in preoperative CMT in patients with microcysts one-month post-surgery. (4) **Conclusions:** There is a relationship between postoperative retinal GCLT and BCVA after uncomplicated cataract surgery. The presence of microcysts significantly reduces the thickness of the GCL, which is significantly related to the loss of BCVA.

## 1. Introduction

Cataracts are the leading cause of blindness in people older than 50 years of age worldwide [1], with an estimated 7 million surgeries per year in Europe and 20 million surgeries per year worldwide [2]. Cataract surgery via the phacoemulsification technique is the most prevalent surgical procedure in medical specialties. The anatomical and functional outcomes are excellent, and complication rates are low. Phacoemulsification alters the blood–aqueous barrier, resulting in postoperative inflammation. Pseudophakic cystoid macular edema (PCME) or Irvine–Gass syndrome is defined as intraretinal fluid in the form of cysts or central macular thickening. It remains the most common cause of decreased visual acuity after uncomplicated phacoemulsification [3]. Historically, PCME is defined with fluorescein angiography, for which the incidence was 9 to 19%. Later, OCT (Optical Coherence Tomography) replaced fluorescein angiography, and the incidence of this disease ranged from 1 to 4%, although cysts could be found in up to 6.39% of patients. Clinically significant PCME can lead to visual acuity impairment and morphologic alterations in OCT; its incidence in the last decade has ranged from 0.02 to 4.2% after uncomplicated phacoemulsification [4]. Macular thickness increases up to 6 months after uncomplicated cataract surgery in both diabetes mellitus (DM) patients without diabetic retinopathy (DR) and non-diabetic subjects [5].

A recently demonstrated risk factor is a central macular thickness >260.5 µm measured with OCT before cataract surgery, which increases the probability of developing intraretinal cysts by 9.08 times [6]. Some studies have demonstrated that alterations of the ganglion cell layer thickness (GCLT), inner nuclear layer (INL), and Muller cells layer are produced in PCME with posterior GCL atrophy.

The ganglion cell is the first-order neuron in the visual pathway, with the cell body located in the retinal GCLT. The thickness of the GCL can be modified by sex and eyeball size [7]. Other studies suggest that the deprivation of amblyopia caused by unilateral congenital or developmental cataracts in children may be associated with GCLT [8], including factors such as age [9], alcoholism, inflammation, and diabetes where the neurodegeneration of inner retinal layers occurs even before the onset of diabetic retinopathy [10], myopia and glaucoma [11], traumatic injuries, retinal detachment with the macula off [12], glaucoma and multiple sclerosis, and have been shown to modify the thickness of the GCLT [13]. Different studies have demonstrated the relationship between cataract surgery by phacoemulsification and changes in the GCLT [14]. Notably, taking into account that the opacification caused by cataracts decreases the OCT signal and can modify the GCLT [15], it has been reported that the GCLT increases slightly after phacoemulsification [16], both in patients without previous ocular pathology and with glaucoma. This increase can be maintained up to 3 months after surgery [17].

At the time, many pathologies that cause macular edema in which a change in GCLT occurs have been reported. Several studies have shown that macular edema, secondary to non-ischemic retinal branch vein occlusion [18,19] and to diabetic retinopathy (DR), experiences a decrease in the thickness of the GCL after the resolution of the edema, causing a decrease in final visual acuity [20]. However, it has been reported that in the acute phase of diabetic macular edema, there is an increase in the thickness of the GCL and outer plexiform layer, accompanied by a decrease in VA secondary to the decrease in the outer layers of the photoreceptors [21,22].

Our study aimed to evaluate whether there was a change in GCT after cataract surgery in patients without previous ocular pathology. At the same time, it is analyzed whether, after phacoemulsification, there is an increase or a decrease in GCL and its relationship with PMCE. Finally, the impact of this change on postoperative visual acuity is analyzed.

## 2. Materials and Methods

It is a prospective cohort study of 174 patients with an indication for cataract surgery evaluated in the Ophthalmology department of the Hospital Universitario de Poniente, Almería, Spain. The data were collected, and the OCT scans were analyzed from November 2017 to December 2018. After verifying that they met the inclusion criteria, 441 patients signed the informed consent form and started the study. Of the total number of patients initially included, we were able to obtain complete data from all examinations in 174 patients, the reason being that they did not return to the clinic after the first month’s examination or that the data were incomplete. This study was approved by the Research Ethics Committee of Almeria (CEIC-AL) with code E.C.21/2017. All patients provided signed informed consent, and the ethical and research precepts complied with the Declaration of Helsinki. The exclusion criteria were as follows: patients with diabetic macular edema, retinal vein occlusion, the use of oral NSAIDs, glaucoma treated with prostaglandins, epiretinal membrane, vitreomacular traction, neovascular membrane, or exudative macular degeneration. The variables analyzed were as follows: demographic data (age, sex, smoking), history (glaucoma, diabetes, glycemia and glycosylated hemoglobin, Hb A1c), and eye examinations, including best-corrected visual acuity (BCVA), cataract type and slit lamp classification; Cirrus OCT (Cirrus HD-OCT; Carl Zeiss Meditec, Dublin, CA, USA) measurements of central macular thickness (CMT); the presence of cysts; and ganglion cell layer thickness (GCL) at one day, one month and three months after surgery. BCVA was measured on a decimal scale and transformed to logMAR (logarithm of the minimum angle of resolution) using the formula logMAR = −log (BCVA decimal scale). All patients were evaluated with Cirrus OCT (Cirrus HD-OCT; Carl Zeiss Meditec, Dublin), and patients with an image quality lower than 7 out of 10 were excluded. OCT images were captured by two independent tests and experienced image reading center examiners (CD, BA). For the repeatability study, examiner 1 captured two consecutive macular cube image sets with a 10–15 min period between each OCT acquisition from each individual eye. For the reproducibility study, examiner 2 captured a second macular cube image set 20–25 min after examiner 1 completed the first examination. The segmentation of retinal and choroidal layers was performed automatically using the device software. In cases of segmentation errors, manual corrections of individual A-scans were performed to fit the boundaries of the compartments of interest (inner limiting membrane; outer layer of RPE)

The cataract classification was determined according to the lens opacity grading system III. Eyes with nuclear opalescence (NO) grades between 1 and 5 were included and subdivided into mild (NO_1_), moderate (NO_2_≤, ≤NO_4_), and dense categories (NO_4_).

The data presented in this study are available as follows: https://data.mendeley.com/datasets/px4ftrthxb/1 (accessed on 2 April 2024).

## 3. Results

One hundred and seventy-four eyes from 174 patients were evaluated, and the study was completed. The mean age was 69.6 years (9.25); 56.9% were women, and 43.1% were men. The preoperative logMAR BCVA was 0.52 (0.18). The most frequent type of cataract was nuclear (71.3%). There was no significant difference in cataract grading scales between men (3.25 (0.64)) and women (3.27 (0.73)). The mean preoperative IOP was 13.74 mmHg (3.16). CMT and GCLT were evaluated in all patients by Cirrus OCT before surgery, and the mean values were 257.75 (20.61) μm and 75.87 (10.17) µm, respectively. There were statistically significant differences in preoperative CMT between men and women, with higher basal thickness in men. There was no difference in the GCLT between males and females. The mean GCLT before surgery was 75.64 (9.65) μm in men and 76.04 (10.61) μm in women. The mean energy-adjusted cumulative dissipated energy (CDE) was 6.76 (6.31) for different cataracts. The mean CDE was 4.50 (0.67) for moderate cataracts and 27.7 (7.10) for patients with hard cataracts, respectively.

One of the qualitative variables analyzed was the presence of diabetes; there were no statistically significant differences between diabetic patients without previous retinal lesions and non-diabetic patients in the GCLT before surgery at one day, one month, or three months. Moreover, there were no statistically significant differences in GCLT between smokers and nonsmokers at any study stage. Table 1 compares the evolution of CMT and GCLT one day, one month, and three months post-surgery and evaluates the existence of a significant correlation with the evolution of BCVA. A statistically significant relationship exists between GCLT and BCVA one month after surgery. Although it does not significantly affect the final BCVA three months after surgery, the initial preoperative values of central macular thickness do not reach the initial values. This correlation is consistent with the statistically significant differences between preoperative and one-month post-surgery GCLT (Table 2).

There are statistically significant differences between preoperative and postoperative GCLT at one and three months. (Table 2). In the case of CMT, there were also significant differences between preoperative and postoperative thickness at one and three months (Table 3). In our results, we observed that, in many cases, the GCLT and CMT remain more significantly increased at three months postoperatively than before surgery (see Table 1)

The appearance of macular cysts was recorded the following day, month, and three months after surgery and was detected in 0 (0%), 15 (8.6%), and 5 (2.9%) of the patients, respectively. We divided the patients according to the presence of microcysts. There were statistically significant differences in the BCVA between patients who had microcysts in the first month compared to those in the third month among those who did not. The appearance of cysts one month after surgery was related to the CMT before cataract surgery; the previous CMT was 273.60 (16.08) µm in patients who subsequently presented microcysts and 256.24 (20.39) µm in patients who did not have cysts (*p* < 0.001). Moreover, this difference in thickness was not present in the GCLT before surgery. There was a statistically significant decrease in the GCLT in patients with microcysts at one month, GCLT was 69.93 (22.41) µm in patients with cysts compared with 80.12 (8.41) µm in patients without cysts (effect size according to Cohen’s d of 10.64). However, there was a statistically significant increase in the CMT, which was 369.33 (88.86) µm in patients with cysts at one month, compared to 267.99 (21.58) µm in patients without cysts at one month (Cohen’s effect size 33.91) (Table 4).

The presence of macular cysts at three months was not significantly related to pre-surgery CMT or GCLT. However, there was a statistically significant relationship between the GCLT on the first day after surgery and the presence of three months post-surgery microcysts.

## 4. Discussion

The present study aimed to detect the change in the GCLT after cataract surgery and the impact of this change on the BCVA. Celik E et al. [15] published a prospective study in 2016 that included 30 eyes without previous pathology that had undergone cataract surgery by phacoemulsification without complications; this resulted in an increase in GCLT at one month post-surgery (89.0 ± 5.3) compared with the preoperative value (85.4 ± 4.4). At the same time, this increase was not correlated with age, axial length, central corneal thickness, operative time, or adequate phacoemulsification time. Our study evaluated CMT and GCLT before surgery, with mean values of 257.75 (20.61) μm and 75.87 (10.17) µm, respectively. Statistically significant differences were found between men and women in the CMT, but there was no difference in the GCLT between genders. The mean GCLT prior to surgery was 75.64 (9.65) μm in men and 76.04 (10.61) μm in women. The mean GCLT increased one month after surgery to 79.09 (10.99) μm, and this increase was maintained at 79.87 (8.04) μm for three months. Sex, diabetes without diabetic retinopathy, smoking status, and glaucoma status did not influence the GCLT at one month or three months after cataract surgery. Roh et al. [18] reported an increase in the GCL after cataract surgery, and it was significantly related to pre-surgery GCLT without finding differences between glaucoma and non-glaucoma patients. Our study reported similar results with a postoperative increase in the GCLT correlated to postoperative BCVA. In the case of CMT, the preoperative thickness was significantly related to microcysts one month after cataract surgery but not to the presence of microcysts three months after cataract surgery. After uncomplicated cataract surgery, macular edema was associated with multiple risk factors [23,24]. Gharbiya M. et al. [25] studied 45 uncomplicated cataract surgeries without pathology. They showed that the maximum increase in CMT and the onset of PMCE occurred between 1 and 2 months after surgery. These results are consistent with our study, where the pre-surgery CMT was 257.75 (20.61) μm, and at one-month post-surgery, there was an increase in the mean CMT to 277.86 (45.29) μm. Nakatani Y. [16] published preoperative and postoperative measurements of cataract GCLT and pRNFL thickness in 53 eyes of 53 patients, 13 of whom had glaucoma. Multivariate analysis showed that the segmentation error in GCLT measurements may influence the measurement of this parameter, the preoperative OCT signal intensity, and the degree of subsequent subcapsular opacity. The most significant increase in thickness occurred in eyes without segmentation error, and the postoperative signal intensity was significantly greater in GCLT measurements. After cataract surgery, all the thickness parameters of the GCLT and pRNFL increased slightly. Changes in signal intensity correlated significantly with changes in pRNFL thickness. However, they were not associated with changes in GCLT, indicating that this increase cannot be explained simply by increased signal intensity. In our study, we excluded OCT images with a quality of less than 7/10, which coincided with the findings of Zhang X. et al. [26]. The higher the image quality was, the greater the reliability and repeatability of the measurement, and we reconfirmed this increase in postoperative GCLT as Nakatani Y. et al. [16] reported with a much larger sample size. Kim H.J. et al. [19] studied 30 case–control patients and reported that the minimum GCLT was lower in the eyes with macular edema than those without and correlated with BCVA in non-ischemic CRVO patients. Zheng Zal. et al. [20], in a case–control study of 40 eyes with retinal branch vein occlusion, observed a decreased GCLT, even after anatomic restoration, and it was associated with BCVA prognosis. Similarly, Bonnin S. et al. [21] evaluated 21 eyes with resolved diabetic macular edema and 21 diabetic eyes without macular edema. The mean GCLT significantly decreased in patients with resolved macular edema (73.8 µm ± 14.3 versus 83.2 µm ±5.9) compared with patients without macular edema. All these studies contribute to the role of GCLT in determining visual prognosis in patients with cystoid macular edema. In this study, as mentioned above, we detected lower GCLT and lower BCVA one month after surgery, with values of 69.93 (22.41) µm in patients with PMCE than in those without PMCE (80.12 (8.41) µm). Collaborating researchers, such as Doncel-Fernandez CJ et al. [6], published a prospective study of 174 patients and demonstrated that a preoperative CMT > 260.5 μm increased the probability of intraretinal cysts one month after cataract surgery compared to that in patients with preoperative CMT < 260.50 μm.

The limitation of our study is the low frequency of PMCE cases. It is complicated to obtain a large number of patients with this complication in prospective studies; a longer follow-up may help us obtain more data on the behavior of GCLT since our study was only three months long. A parameter that would be interesting to study not included in this study would be macular vascular perfusion and its impact after cataract surgery measured by optic coherence tomography angiography (OCT-A). Other OCT-A-derived parameters, such as foveal avascular zone (FAZ) parameters like the FAZ area or circularity index, could be studied in a future study. Gawęcki et al. [27], in a retrospective study on 44 patients after uncomplicated cataract surgery, concluded that cataract surgery resulted in an increase in retinal thickness and volume in the first few months after the surgery, followed by a spontaneous decline in these parameters in the subsequent months, and a long-standing improvement was noted in the vessel perfusion full parameter. Baldascino et al. [28], in a cross-sectional retrospective study on two eyes, reported an increase in macular superficial capillary plexus vessel density and perfusion after uncomplicated cataract surgery regardless of the cataract severity. Therefore, macular perfusion is an interesting parameter that may be related to GCLT and can be studied in the future.

The other significant limitation is the measurement of the GCLT using Cirrus OCT as the following indicates: Segmentation Errors: automated segmentation algorithms used by Cirrus OCT can sometimes misidentify or incorrectly segment the GCLT, especially in eyes with pathologies such as macular degeneration, glaucoma, or high myopia. These errors can lead to inaccurate thickness measurements. Artifact Presence: artifacts caused by factors like eye movement, poor fixation, or media opacities (e.g., cataracts) can affect the quality of the OCT images, leading to unreliable GCL measurements. Inter- and Intra-Observer Variability: although automated, manual intervention is sometimes required to correct segmentation errors, it introduces variability depending on the experience and judgment of the observers. Limited Retinal Area Coverage: Cirrus OCT typically scans a predefined area of the retina. If pathology exists outside this area, it may not be detected, leading to incomplete assessment of the GCLT. Device-Specific Normative Data: The normative database used by Cirrus OCT is device-specific. Differences in demographics, ocular characteristics, or underlying conditions in the population from which normative data are derived can affect the generalizability of the results. Depth Resolution Limitations: The axial resolution of Cirrus OCT, although high, has its limitations. Thin retinal layers like the GCL can be challenging to distinguish accurately, particularly in retinal edema or atrophy. Evolving Technology: OCT technology and algorithms are continually evolving. Older models of Cirrus OCT may not have the latest advancements in image processing and segmentation, affecting measurement accuracy compared to newer devices. Assumption of Layer Consistency: the algorithms assume a consistent layer structure, which may not hold true in diseased eyes where retinal layers can be disrupted or abnormally thickened/thinned.

By acknowledging these limitations, we can better interpret GCL measurements from Cirrus OCT and consider repeated measures to confirm our findings or complementary diagnostic tools.

## 5. Conclusions

This study shows the relationship between the postoperative increase in the retinal ganglion cell layer thickness and BCVA after uncomplicated cataract surgery. However, the presence of microcysts reduces the thickness of the GCL, which is significantly related to the loss of BCVA.

## Figures and Tables

**Table 1:** Evolution of OCT parameters and correlation with BCVA.

*p*-Value Pearson Correlation of BCVA (logMAR)						
	Mean (µm)	SD (µm)	Preoperative	1-Day	1-Month	3-Months
Preop GCLT	75.87	10.17	0.934	0.903	0.984	0.312
Preop CMT	257.75	20.61	0.453	0.709	0.596	0.627
1-day GCLT	79.51	44.29	0.406	0.955	0.376	0.611
1-day CMT	255.62	19.56	0.72	0.707	0.296	0.705
1-month GCLT	79.09	10.99	0.341	0.159	0.040 *	0.221
1-month CMT	277.86	45.29	0.506	0.296	0.001 *	0.705
3 months GCLT	79.87	8.04	0.887	0.237	0.788	0.245
3 months CMT	267.86	20.17	0.571	0.904	0.346	0.648

* *p* < 0.05.

**Table 2:** GCLT paired sample test.

GCLT	Paired Differences					t	df	Significance	
	Mean	Std. Dev	SE Mean	95% CI				One-Sided *p*	Two-Sided *p*
				Lower	Upper				
Preop-1 day	−3.306	45.823	4.115	−11.452	4.839	−0.8	123	0.212	0.423
Preop-1 month	−3.467	12.129	1.036	−5.516	−1.418	−3.34	136	0.001	0.001
Preop-3 month	−3.453	6.774	0.658	−4.757	−2.148	−5.24	105	<0.001	<0.001
1 day-1 month	0.402	47.32	4.471	−8.458	9.262	0.09	111	0.464	0.929
1–3 months	−0.5	9.195	0.902	−2.288	1.288	−0.55	103	0.29	0.58

CMT = central macular thickness (µm), Std. Dev = standard deviation, SE = standard error, and CI = confidence intervals.

**Table 3:** CMT paired sample test.

CMT	Paired Differences					t	df	Significance	
	Mean	Std. Dev	SE Mean	95% CI				One-Sided *p*	Two-Sided *p*
				Lower	Upper				
Preop-1 day	2.233	12.441	1.136	−0.015	4.482	1.967	119	0.026	0.052
Preop-1 month	−19.961	39.923	3.217	−26.317	−13.605	−6.205	153	<0.001	<0.001
Preop-3 month	−10.285	12.851	1.022	−12.304	−8.265	−10.059	157	<0.001	<0.001
1 day-1 month	−20.657	36.665	3.528	−27.651	−13.663	−5.855	107	<0.001	<0.001
1–3 months	5.951	24.314	2.04	1.917	9.984	2.917	141	0.002	0.004

CMT = central macular thickness (µm), Std. Dev = standard deviation, SE = standard error, and CI = confidence intervals.

**Table 4:** Relationship between the presence of one-month post-surgery microcysts and OCT variables.

Group Statistics						t	df	Significance		Mean Difference	SE Difference	95% CI	
		N	Mean	Std. Dev	SE Mean			One-Sided *p*	Two-Sided *p*			Lower	Upper
Preop GCLT	No	150.00	75.79	10.44	0.85	−0.33	162.00	0.37	0.75	−0.93	2.85	−6.55	4.70
	Yes	14.00	76.71	6.79	1.81								
Preop CMT	No	158.00	256.24	20.39	1.62	−3.89	18.56	0.00	0.00	−17.36	4.46	−26.70	−8.02
	Yes	15.00	273.60	16.08	4.15								
One-day GCLT	No	112.00	75.88	9.43	0.89	−0.93	11.01	0.19	0.37	−37.45	40.48	−126.54	51.64
	Yes	12.00	113.33	140.20	40.47								
One day CMT	No	108.00	253.43	18.96	1.82	−3.89	118.00	0.00	0.00	−21.91	5.63	−33.05	−10.76
	Yes	12.00	275.33	13.19	3.81								
One-month GCLT	No	124.00	80.12	8.41	0.76	3.42	136.00	0.00	0.00	10.19	2.98	4.29	16.09
	Yes	14.00	69.93	22.41	5.99								
One-month CMT	No	139.00	267.99	21.58	1.83	−4.40	14.18	0.00	0.00	−101.34	23.02	−150.65	−52.03
	Yes	15.00	369.33	88.86	22.94								

CMT = central macular thickness (µm), GCLT = ganglion cell layer (µm), Std Dev = standard deviation, and SE = standard error.

## Data Availability

The data presented in this study are available as follows: Castro de Luna, Gracia (2023), “Retinal ganglion cell and cataract surgery”, Mendeley Data, V1, https://data.mendeley.com/datasets/px4ftrthxb/1 (accessed on 2 April 2024).
